# Mild hypoglycemia is strongly associated with increased intensive care unit length of stay

**DOI:** 10.1186/2110-5820-1-49

**Published:** 2011-11-24

**Authors:** James Krinsley, Marcus J Schultz, Peter E Spronk, Floris van Braam Houckgeest, Johannes P van der Sluijs, Christian Mélot, Jean-Charles Preiser

**Affiliations:** 1 Division of Critical Care, Stamford Hospital, Columbia University College of Physicians and Surgeons, Stamford, CT, USA; 2 Department of Intensive Care, Academic Medical Center, University of Amsterdam, Amsterdam, The Netherlands; 3 Laboratory of Experimental Intensive Care and Anesthesiology (L·E·I·C·A), Academic Medical Center, University of Amsterdam, Amsterdam, The Netherlands; 4 Department of Intensive Care, Gelre Hospitals, location Lukas, Apeldoorn, The Netherlands; 5 Department of Intensive Care, Tergooi Hospitals, location Blaricum, Blaricum, The Netherlands; 6 Department of Intensive Care Medicine, Medical Center Haaglanden, The Hague, The Netherlands; 7 Department of Intensive Care, Erasme University Hospital, Brussels, Belgium; 8 Department of Emergency Medicine, Erasme University Hospital, Brussels, Belgium

**Keywords:** hypoglycemia, intensive care unit, length of stay, resource utilization, APACHE II, mortality, intensive insulin therapy

## Abstract

**Background:**

Hypoglycemia is associated with increased mortality in critically ill patients. The impact of hypoglycemia on resource utilization has not been investigated. The objective of this investigation was to evaluate the association of hypoglycemia, defined as a blood glucose concentration (BG) < 70 mg/dL, and intensive care unit (ICU) length of stay (LOS) in three different cohorts of critically ill patients.

**Methods:**

This is a retrospective investigation of prospectively collected data, including patients from two large observational cohorts: 3,263 patients admitted to Stamford Hospital (ST) and 2,063 patients admitted to three institutions in The Netherlands (NL) as well as 914 patients from the GLUCONTROL trial (GL), a multicenter prospective randomized controlled trial of intensive insulin therapy.

**Results:**

Patients with hypoglycemia were more likely to be diabetic, had higher APACHE II scores, and higher mortality than did patients without hypoglycemia. Patients with hypoglycemia had longer ICU LOS (median [interquartile range]) in ST (3.0 [1.4-7.1] vs. 1.2 [0.8-2.3] days, *P *< 0.0001), NL (5.2 [2.6-10.3] vs. 2.0 [1.3-3.2] days, *P *< 0.0001), and GL (9 [5-17] vs. 5 [3-9] days, *P *< 0.0001). For the entire cohort of 6,240 patients ICU LOS was 1.8 (1.0-3.3) days for those without hypoglycemia and 3.0 (1.5-6.7) days for those with a single episode of hypoglycemia (*P *< 0.0001). This was a consistent finding even when patients were stratified by severity of illness or survivor status. There was a strong positive correlation between the number of episodes of hypoglycemia and ICU LOS among all three cohorts.

**Conclusions:**

This multicenter international investigation demonstrated that hypoglycemia was consistently associated with significantly higher ICU LOS in heterogeneous cohorts of critically ill patients, independently of severity of illness and survivor status. More effective methods to prevent hypoglycemia in these patients may positively impact their cost of care.

## Introduction

Hyperglycemia occurs commonly in critically ill patients and is strongly associated with increased risk of mortality [1-3]. During the past decade, a number of interventional trials have assessed the impact of intensive insulin therapy (IIT) to correct even moderate degrees of hyperglycemia; several have resulted in improvements in mortality and/or morbidity [4-6], whereas a number did not demonstrate benefit [7-11]. Hypoglycemia, either spontaneous or occurring as a complication of IIT, is a frequent occurrence in critically ill patients and is independently associated with increased risk of mortality [12-15]. Whereas severe hypoglycemia, usually defined as blood glucose level (BG) < 40 mg/dL, has been the focus of most of these studies [9,12-16], other investigators have demonstrated a deleterious impact of even mild hypoglycemia--BG < 70 mg/dL--on survival in heterogeneous populations of critically ill patients [17,18].

The cost of treating intensive care unit (ICU) patients is enormous. It has been estimated that 0.5-1.0% of the United States Gross Domestic Product is consumed in the ICU, representing 20-30% of a typical hospital's costs [19,20]. A limited body of literature has explored the impact of glycemic management protocols on the cost of care in ICU populations [21-24]. These data suggest that significant cost savings accrue from amelioration of hyperglycemia in the critically ill, associated with reductions in ICU length of stay (LOS), ICU acquired infections, and decreases in pharmacy, laboratory, and diagnostic imaging use. To date, however, no studies have investigated the impact of hypoglycemia on the cost of care of critically ill patients.

The purpose of this study was to evaluate the impact of hypoglycemia, defined as BG < 70 mg/dL, on resource utilization in the ICU. The choice of a threshold value of 70 mg/dL was based on several factors. Cryer has detailed the pathophysiologic consequences of hypoglycemia defined at this threshold [25]. Moreover, two recent observational cohort studies have demonstrated an independent association of mild hypoglycemia with mortality [17,18]. Consequently, we hypothesized that hypoglycemia would impact the magnitude of resource utilization, reflected by ICU LOS.

We have the unique opportunity to analyze a large diverse group of critically ill patients in this international collaboration; the 3 datasets include a large single-center cohort from an ICU in the United States (Stamford Hospital), 3 ICUs from The Netherlands, and 21 ICUs from Western Europe and Israel that participated in the GLUCONTROL trial, a multicenter randomized controlled trial of intensive insulin therapy [8].

## Methods

### Settings, patients, glycemic control programs and data accrual

The Stamford cohort. Stamford Hospital is a 305-bed, university-affiliated hospital. The 16-bed adult ICU treats a heterogeneous population of medical, surgical, and trauma patients. Medical and surgical house staff, closely supervised by a team of intensivists, delivers care. The patient cohort in Stamford (ST) includes 3,263 patients admitted to the ICU between January 12, 2007 and April 30, 2010 who had at least three blood glucose values obtained during their ICU stay. Forty-one patients admitted during this period with a diagnosis of diabetic ketoacidosis or hyperosmolar nonketotic coma were excluded from the study. The glycemic target during the period of the investigation was 80-125 mg/dL. Details of the protocol have been published previously [26]. Most of the BG measurements (85%) were made using bedside glucometers (AccuChek Inform, Indianapolis, IN) and capillary or venous blood; the remainder were performed in the central laboratory using a using a Siemens Advia 1800 analyzer (Siemens Medical Solutions, Malvern, PA) or in the ICU using a GEM4000 point of care analyzer (Instrument Laboratory, Lexington, MA). Data were abstracted from the ICU's comprehensive clinical database. Diabetic status was determined prospectively based on all available clinical information at the time of ICU admission.

The Dutch cohort. The three hospitals in The Netherlands are university-affiliated hospitals, with 700 beds (Gelre Hospital, Apeldoorn, The Netherlands), 633 beds (Tergooi Hospitals, Hilversum, The Netherlands), and 785 beds (Medical Center Haaglanden, The Hague, The Netherlands). The 10-bed, 9-bed, and 18-bed adult ICUs treat a heterogeneous population of medical, surgical, and trauma patients. A team of intensivists delivers care in a closed-format setting. The patient cohort in The Netherlands (NL) includes 2,063 patients admitted to the ICU between January 1, 2007 and December 29, 2009, who had at least three blood glucose values obtained during their ICU stay: 1,098 patients (NL-L) admitted between January 1, 2007 and January 31, 2008 were subjected to a "loose" intensive insulin therapy guideline, and 965 patients (NL-S) admitted between February 1, 2008 and December 29, 2009 were subjected to a "strict" intensive insulin therapy guideline (see below for details on "loose" and "strict" glucose control). Per protocol, patients admitted during this period with a diagnosis of diabetic ketoacidosis or hyperosmolar nonketotic coma were not subjected to treatment according to the guideline. Loose intensive insulin therapy: blood glucose control in the three participating ICUs followed the 2004 Surviving Sepsis Campaign Guidelines [27,28] and aimed for a BG < 150 mg/dl. Insulin dose and route of administration (intravenous or subcutaneous) and timing and type of blood glucose measurement (using capillary or arterial blood, at the bedside or in a central laboratory) were loosely defined in the guidelines in use. ICUs nurses practiced blood glucose control. Strict intensive insulin therapy: blood glucose control in the three participating ICUs aimed for a BG between 80-110 mg/dl; administration of insulin was intravenous at all times, and BG measurements were performed at the bedside. Blood glucose control required a high level of intuitive decision-making. All BG measurements were made by using bedside glucometers (AccuChek Inform; Roche, Almere, The Netherlands) and arterial blood. Details of the protocol have been published previously [18]. Data were abstracted from the National Intensive Care Evaluation (NICE) database, created daily by the responsible intensivists (PES, FvBH, JPvdS) and maintained by the NICE Foundation [29].

The GLUCONTROL cohort. This cohort included data from patients enrolled in the GLUCONTROL trial [8] in 1 of the 21 units from 19 different hospitals in 7 different countries of Western Europe and Israel, between November 3, 2004 and May 30, 2006. The number of ICU beds of the participating units ranged from 5 to 44 (median, 12). Patients were randomized to an intensive insulin therapy (target BG: 80-110 mg/dl) (GL-IIT) or to a control arm (GL-C) with an intermediate glucose target (140-180 mg/dl), using an insulin protocol. BG checks were performed on arterial or central venous samples when a catheter was in place and a blood gas analyzer was preferentially used. Capillary samples and a specific glucometer (Accu-Check Inform, Roche Diagnostics, Mannheim, Germany) were allowed. The data from the 914 patients with at least three BG checks and survivor status were analyzed; the other 164 patients were evenly distributed between the GL-IIT and GL-C groups (n = 82 in each arm).

Additional details about the glycemic control protocols used in the three cohorts can be found in a recent publication [18].

### Statistical analysis

Continuous data are presented as mean (standard deviation) or median (interquartile range), as appropriate, and compared by using Student's *t *test or the Mann-Whitney rank-sum test, respectively. Categorical data are presented as percentages and compared using the Chi-square test. Multivariate analysis to assess the independent association of any hypoglycemia (BG < 70 mg/dL), as well as BG < 50 mg/dL and 50-69 mg/dL, with ICU LOS included the following parameters found to be statistically significant at *P *< 0.1 on univariate analysis: age, modified APACHE II score (age component deleted to avoid colinearity with age in the multivariate analysis: age 45-54, 2 points; age 55-64, 3 points; age 65-74, 5 points; age ≥ 75, 6 points), medical diagnostic category on admission to the ICU and mechanical ventilation. The same model was used to assess independent contributors to the risk of prolonged ICU stay, defined as greater than the 75^th ^percentile for each cohort (3.1, 6.6, and 12.8 days for the ST, NL, and GL cohorts, respectively). Diabetes was not associated with mortality on univariate analysis and therefore was not entered into the multivariate model.

Mortality is defined throughout as hospital, not ICU, mortality. Statistical analysis was performed using the MedCalc statistical package version 10.1.1.6.0 http://www.medcalc.be.

## Results

### Characteristics of the patients

In brief, age (all five subpopulations) and diabetic status (data not available for NL cohorts) were similar. The percentage of patients with nonsurgical admitting diagnoses ranged from 39.9% (GL-C) to 64.1% (NL-L). Mean (SD) APACHE II scores ranged from 16.0 (9.0) (ST) to 19.7 (8.2) (NL-L), and mortality ranged from 14.2% (ST) to 27.5% (NL-L).

Significant differences in glycemic control also were noted [16]. The median (IQR) number of BG measurements per day ranged from 5.1 (3.6-7.6) (NL-L) to 9.3 (8.0-11.3) (ST). Mean BG (median, [IQR]) ranged from 117.9 (107.0-137.0) (NL-S) to 146.3 (128.1-164.6) (GL-C) and coefficient of variation (CV, %) (median [IQR]) from 21.0 (14.8-28.5) (ST) to 31.8 (23.8-40.8) (NL-S). Finally, the percentage of patients who experienced at least one episode of hypoglycemia (BG < 70 mg/dL) ranged from 17.8% (GL-C) to 64.9% (NL-S)

### Comparison of patients with and without hypoglycemia

Table 1 demonstrates differences between patients with hypoglycemia, including patients with minimum BG < 70 mg/dL, 50-69 mg/dL, and < 50 mg/dL, and those without hypoglycemia for the entire cohort of 6,240 patients. Patients with hypoglycemia were older, more likely to be admitted to the ICU with a nonsurgical diagnosis, and more likely to be diabetic. They had higher APACHE II scores and higher mortality. Additional differences included lower mean BG concentrations and higher CV.

**Table 1:** Comparison of patients with hypoglycemia to those without hypoglycemia

	Minimum BG < 70 mg/dL	Minimum BG 50-69 mg/dL	Minimum BG < 50 mg/dL	Minimum BG ≥ 70 mg/dL
Number	2,313	1,424	889	3,927
Age (yr)	70 (57-79)	70 (59-80)	69 (58-78)	66 (52-78)
DM (%)*	27.3	28.3	26.8	17.5
MED patient (%)	56.2	56.0	56.6	54.7
ICU LOS	5 (2.2-10.5)	4.2 (2-9)	6 (2.8-12.2)	1.8 (1.0-3.3)
APACHE II	20.8 (8.4)	19.9 (8.1)	22.2 (8.8)	15.2 (8.1)
Mortality (%)	29.6	26.5	34.6	13.1
Glucose control				
BG per patient	45 (21-97)	36 (18-78)	65 (29-127)	11 (7-24)
BG per day	9.5 (7.2-11.9)	9.2 (6.6-11.2)	10.2 (8.1-12.5)	8 (5-10)
Mean (mg/dL)	118.3 (108.1-132.5)	120.0 (109.3-133.4)	116.5 (106.5-129.4)	128.1 (115.3-144.4)
CV (%)	31.6 (25.0-40.0)	29.0 (23.1-37.4)	35.0 (29-43.7)	19.2 (13.7-26.1)

*Includes only patients from ST and GL cohorts. Data displayed as percentage, median (interquartile range), or mean (standard deviation). P values comparing patients without hypoglycemia to those with minimum BG < 70 mg/dL < 0.0001, except for MED patient p = 0.2581. DM, diabetes mellitus; MED patient, medical diagnosis on admission to the ICU, rather than surgical or trauma; BG, blood glucose; BG per patient, number of BG measurements per patient; BG per day, number of BG measurements per day per patient; Mean, individual patient's mean BG during ICU stay; CV, individual patient's mean coefficient of variation during ICU stay.

**Figure 1:**
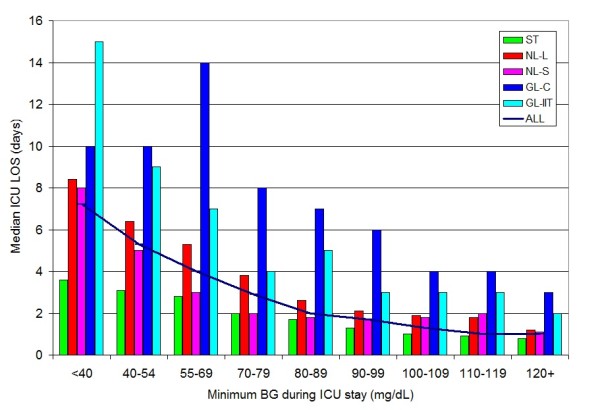
**Relationship between minimum BG during ICU stay and ICU LOS: 3 cohorts**.

Figure 1 illustrates the negative correlation between minimum BG during ICU stay and ICU LOS for the different cohorts (*P *for trend < 0.0001 for each of the cohorts).

### Multivariate analysis of factors associated with ICU LOS

Table 2 demonstrates that hypoglycemia--minimum BG < 50 mg/dL as well as minimum BG 50-69 mg/dL--is independently associated with prolonged ICU LOS, defined as greater than the 75^th ^percentile for each cohort (3.1, 6.6, and 12.8 days, respectively, for ST, NL, and GL).

**Table 2:** Multivariate analysis of factors independently associated with prolonged ICU LOS

	OR (95% CI)	*P *value
Mechanical ventilation	3.82 (3.20-4.51)	< 0.0001
Minimum BG < 70 mg/dL	2.50 (2.12-2.95)	< 0.0001
Minimum BG 50-69 mg/dL	2.16 (1.81-2.59)	< 0.0001
Minimum BG < 50 mg/dL	1.78 (1.39-2.29)	< 0.0001
Medical diagnosis on admission	1.59 (1.34-1.88)	< 0.0001
Modified APACHE II score	1.04 (1.03-1.06)	< 0.0001
Age	1.00 (0.99-1.01)	0.6002

The 5 parameters in these multivariate models were each significant at p < 0.10 on univariate analysis. Diabetes was not significant on univariate analysis and is therefore not included in the models. Prolonged ICU LOS is defined as greater than the 75^th ^percentile for each cohort (3.1, 6.6, and 12.8 days for ST, NL and GL respectively). OR per year for age and per point for Modified APACHE II score.

### Association between ICU LOS and hypoglycemia, stratified by severity of illness survivor status

**Figure 2:**
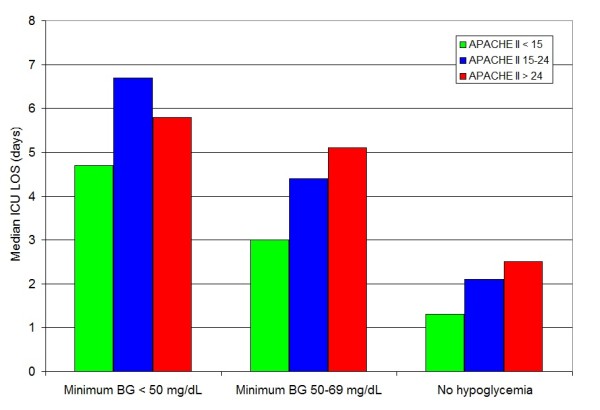
**Relationship between hypoglycemia and ICU LOS, stratified by APACHE II score**.

**Figure 3:**
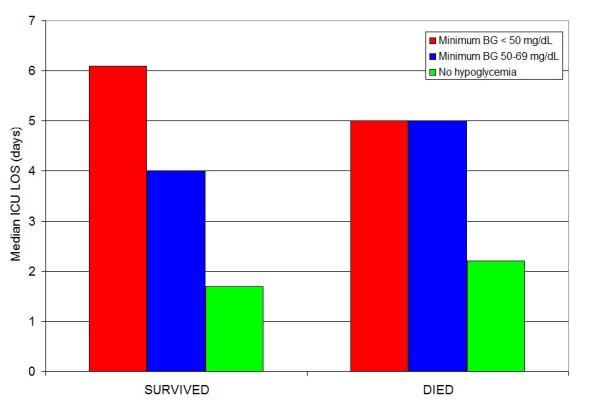
**Relationship between hypoglycemia and ICU LOS, stratified by survivor status**.

Figure 2 illustrates that the difference in ICU LOS comparing patients with hypoglycemia and patients without hypoglycemia is found across different ranges of severity of illness, reflected by admission APACHE II score. Figure 3 stratifies this relationship by survivor status.

### Dose response relationship between hypoglycemia and ICU LOS

**Figure 4:**
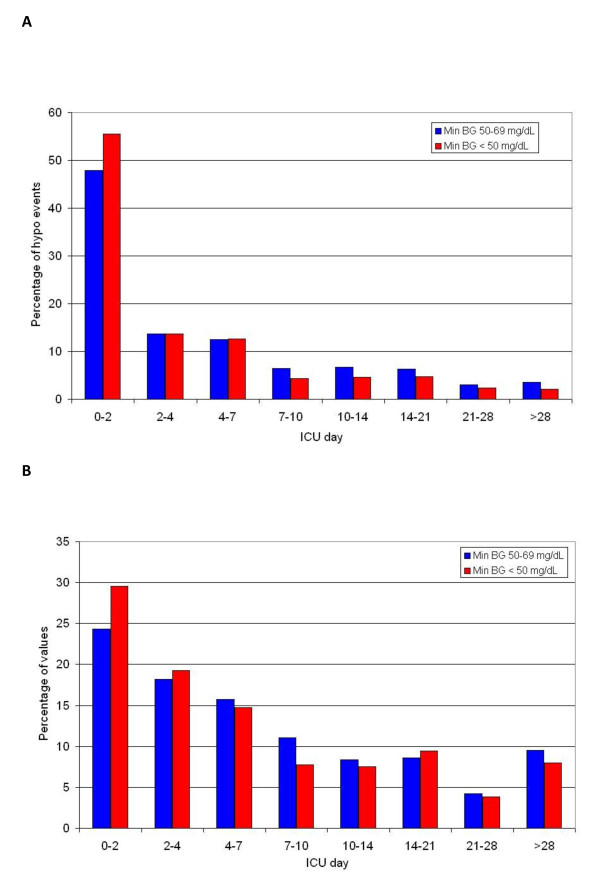
**Timing of hypoglycemic events**. (a) Timing of hypoglycemic events: Stamford cohort. (b) Timing of hypoglycemic events: Netherlands cohort.

**Figure 5:**
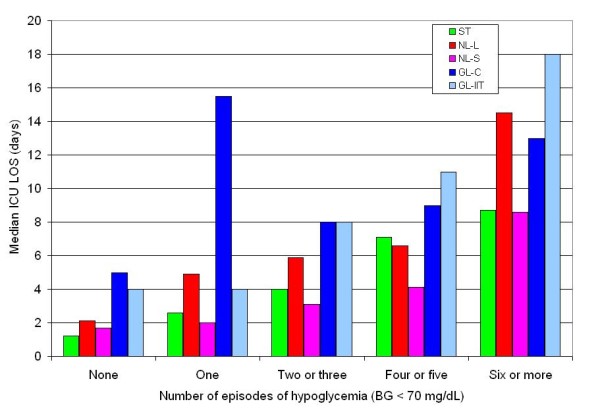
**Relationship between number of episodes of hypoglycemia and ICU LOS: 3 cohorts**.

Figures 4a and 4b illustrate that most hypoglycemic events occurred soon after ICU admission for patients in the ST and NL cohorts. Of the patients with hypoglycemia in the ST cohort, 47% cohort had an episode within the first 48 hours of ICU admission; in the NL cohort, 72% of the patients with hypoglycemia in the NL cohort had an episode within the first 48 hours of ICU admission. Figure 5 displays the strong association between the number of episodes of hypoglycemia and ICU LOS. For the entire cohort of 6,240 patients, ICU LOS was 1.8 (1.0-3.3) days for those without hypoglycemia (n = 3,917) and 3.0 (1.5-6.7) days for those with a single episode of hypoglycemia (n = 774; *P *< 0.0001).

## Discussion

Although emerging literature has documented the strong association between hypoglycemia during acute and critical illness and an increased risk of mortality [9,11-18], this is the first investigation that has focused explicitly on the association of hypoglycemia with ICU LOS, the predominant driver of resource utilization in this population. The salient finding of this investigation is that patients sustaining even a single episode of BG < 70 mg/dL during ICU stay incurred substantially greater LOS than did those without an episode of hypoglycemia: 1.8 (1.0-3.3) vs. 3.0 (1.5-6.7) days (*p *< 0.0001). This observation was independent of survivor status or severity of illness, as reflected by admission APACHE II score. The relationship between ICU LOS and hypoglycemia was remarkably consistent in these three separate cohorts of patients. Finally, there was a dose response relationship between hypoglycemia and resource utilization: the number of discrete episodes of hypoglycemia was directly and positively correlated with ICU LOS.

The major interventional trials of IIT [4-8,10] as well as large observational cohort studies [12,13,16,17] describing the association of hypoglycemia with mortality do not detail differences in ICU LOS comparing those who experienced hypoglycemia to those who did not. However, the findings of the current investigation corroborate the limited data available in the literature that do address this topic. Arabi et al. analyzed severe hypoglycemic events (BG < 40 mg/dL) that occurred in their randomized, controlled trial of IIT [9]. ICU LOS (median, IQR) was considerably longer in patients with hypoglycemia than in those without: 5.8 (2.0-12.9) vs. 1.0 (0.8-1.9) (*p *value not supplied). Additionally, Vriesendorp and colleagues performed an observational cohort study of patients sustaining severe hypoglycemia (BG < 45 mg/dL) [15]. Index cases and controls were matched by the time of the hypoglycemic event. The median (range) time in days from the index moment to death or hospital discharge was longer in patients with hypoglycemia: 11 (0-204) vs. 8 (0-146; *p *value not provided).

The multicenter, international nature of the investigation increases the generalizability of the findings; the heterogeneous 6,240 patient cohort were admitted with varying severities of illness and ICU LOS and treated in ICUs using different glycemic targets, measurement technologies, and glycemic management protocols. One limitation is the absence of data differentiating between spontaneous and therapy-induced hypoglycemia; it is unclear whether these may have the same association with increased ICU LOS. The use of bedside glucometers for measurement of capillary blood is an additional limitation of this investigation, because this measurement technology has been associated with analytic inaccuracies, especially in the hypoglycemic range [30-32]. Notably, the retrospective nature of this investigation is an acknowledged weakness. This was unavoidable, because it would be unethical to perform a randomized, controlled trial of induced hypoglycemia in a population of critically ill patients. However, while the design of the study precludes proof of causality, there are several lines of evidence that suggest strongly that hypoglycemia led to increased resource utilization, rather than was a consequence of more frequent BG measurements in patients who required longer ICU stays. Hypoglycemia occurred early in the course of ICU stay; 47% and 72% of the patients with hypoglycemia in the ST and NL cohorts, respectively, experienced an episode within the first 48 hours of ICU stay. Moreover, the relationship between hypoglycemia and increased LOS was independent of severity of illness; patients with hypoglycemia who had mild, moderate, or severe levels of illness, reflected by APACHE II score sustained significantly longer LOS than did those without hypoglycemia, and this relationship was seen for survivors as well as nonsurvivors.

There are some possible links between hypoglycemia and worsened outcome or complicated course of critical illness [25,33]. First, the physiological mechanisms triggered by hypoglycemia are commonly impaired during critical illness. These include the inhibition of insulin release, typically occurring when BG is < 80 mg/dl, an increased release of glucagon, epinephrine, and growth hormone when BG is < 65 mg/dl, and increase release of cortisol when BG is < 55 mg/dl [34]. During critical illness, exogenous insulin is infused and the levels of glucagon, epinephrine, cortisol, and growth hormone are typically already elevated. Second, large swings in BG, as observed when hypoglycemia is aggressively treated with a large amount of intravenous glucose, are typically associated with cellular damage [35]. Third, the detrimental effects of hypoglycemia are well documented in the brain. Indeed, glucose is the preferential energetic substrate in the brain. The absence of cerebral stores of glucose and the diffusive character of transport imply that the glucose concentration in neurons and glial cells is entirely determined by BG [35].

The main driver of the cost of care of patients admitted to the ICU is length of stay [21,22,36]. This investigation, demonstrating consistent evidence of increased ICU LOS among critically ill patients sustaining hypoglycemia compared with those without hypoglycemia, has important implications for the management of these patients. Although this study must be considered hypothesis-generating, the evidence from this study and other recent investigations strongly suggests that avoidance of hypoglycemia has a beneficial effect not only on survival, but on cost, an important goal in the context of estimates that ICU care consumes 20-30% of individual hospital's resources and 0.5-1.0% of US Gross National Product [19,20].

## Conclusions

This multicenter investigation demonstrates a strong association between mild hypoglycemia (BG < 70 mg/dL) and increased ICU LOS, independent of severity of illness and survivor status. Successful avoidance of hypoglycemia has the potential to significantly decrease the cost of care of the critically ill.

## Abbreviations

APACHE II: Acute Physiology and Chronic Health Evaluation II; BG: blood glucose concentration (mg/dL); CV: coefficient of variation; DM: diabetes mellitus; GL: GLUCONTROL cohort; ICU: intensive care unit; LOS: length of stay; NL: Netherlands cohort; SD: standard deviation; ST: Stamford cohort.

## Competing interests

James S. Krinsley, MD, has performed consulting work for Medtronic Inc., Edwards Life Sciences, Baxter, Roche Diagnostics, and Optiscan Biomedical and has received speaker's fees from Edwards Life Sciences, Roche Diagnostics and Sanofi-Aventis. Marcus J. Schultz, MD, PhD, has performed consulting work for Medtronic Inc. and Optiscan Biomedical and has received research support from Optiscan Biomedical. Peter E. Spronk, MD, PhD, FCCP, Floris van Braam Houckgeest, MD, Johannes P. van der Sluijs, MD, PhD, and Christian Mélot, MD, PhD, have no disclosures to report. Jean-Charles Preiser, MD, PhD, has performed consulting work for Medtronic Inc., Edwards Life Sciences, and Optiscan Biomedical.

## Authors' contributions

JK wrote the initial and subsequent drafts of the manuscript and performed statistical analysis. MS reviewed all drafts of the manuscript and assisted with revisions. PS, FH, JS, and CM helped with data collection and reviewed the drafts of the manuscript. JCP reviewed all drafts of the manuscript and assisted with revisions. All authors read and approved the final manuscript.

This investigation was not supported by any source of external funding.
